# Supplementary figures and images for: DksA-dependent regulation of RpoS contributes to Borrelia burgdorferi tick-borne transmission and mammalian infectivity
Source: PLoS Pathog. 2021 Feb 18;17(2):e1009072. doi: 10.1371/journal.ppat.1009072 (PMC7924775; doi:10.1371/journal.ppat.1009072)

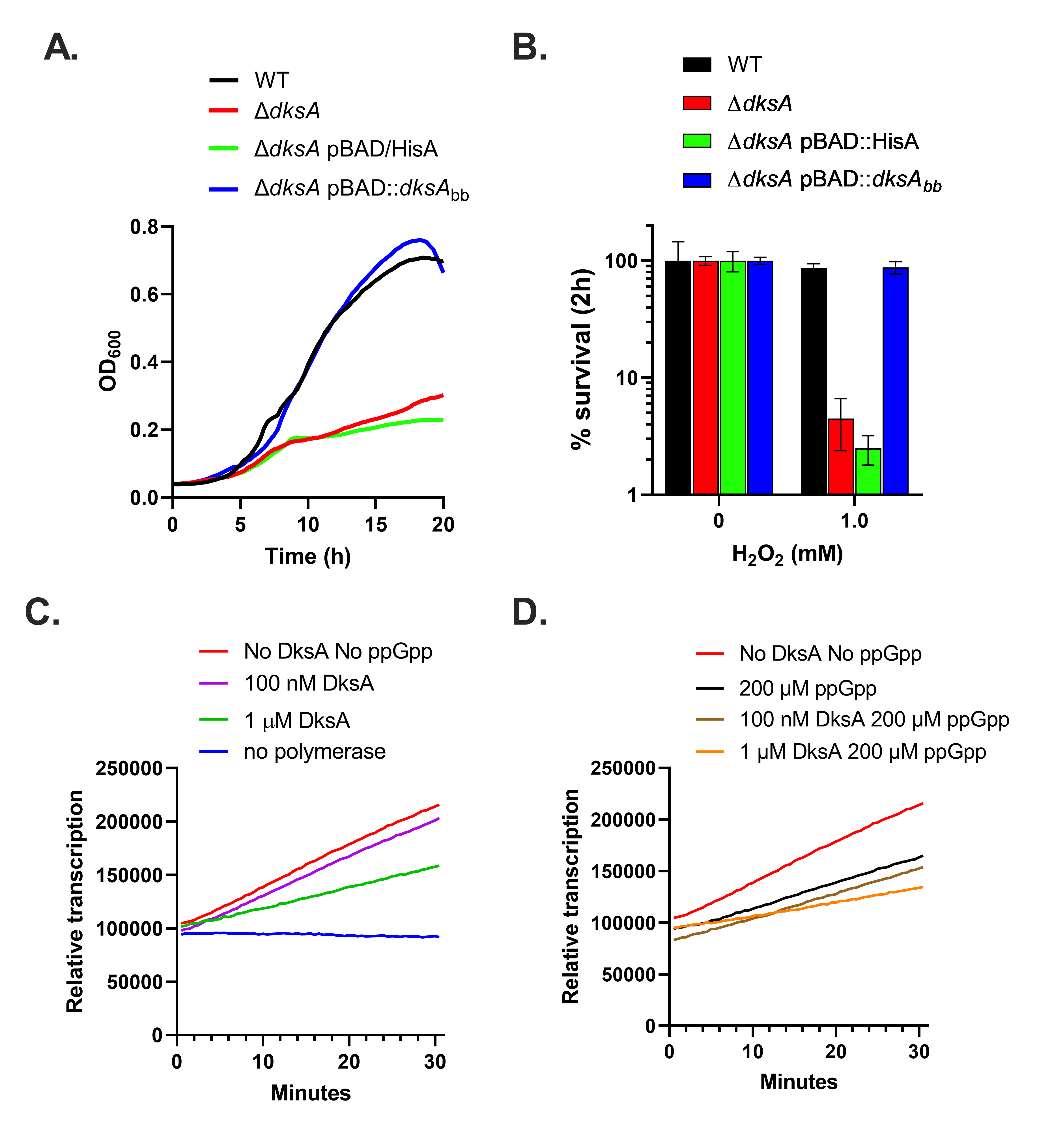

Supplement: S1 Fig — The growth of wild type, ΔdksA, ΔdksA pBAD (empty vector control), and ΔdksA pBAD::dksAbb was compared in minimal N salts medium supplemented with 0.5 mM MgCl2, 0.1% casamino acids, and 0.02% L-arabinose (A). Data represent represent 3 independent experiments. The susceptibility to killing of E. coli strains by 1.0 mM H2O2 was determined after 2 h of incubation at 37°C in N salts minimal medium (B). The data represent the mean ± S.D. of 4 replicates from 2 separate experiments. Repression of in vitro transcription from the E. coli rrnB P1 promoter by B. burgdorferi DksA and ppGpp (C and D). E. coli RNA polymerase transcription from rrnB P1 was detected real-time by a molecular beacon assay. Reactions containing no B. burgdorferi DksA or ppGpp produce a higher rate of transcription compared to those contain DksA and ppGpp. Data represent 2 replicate experiments. DksA and ppGpp are identical stocks to those used in subsequent experiments (Figs 2 and 3). (TIF) [file ppat.1009072.s001.tif]

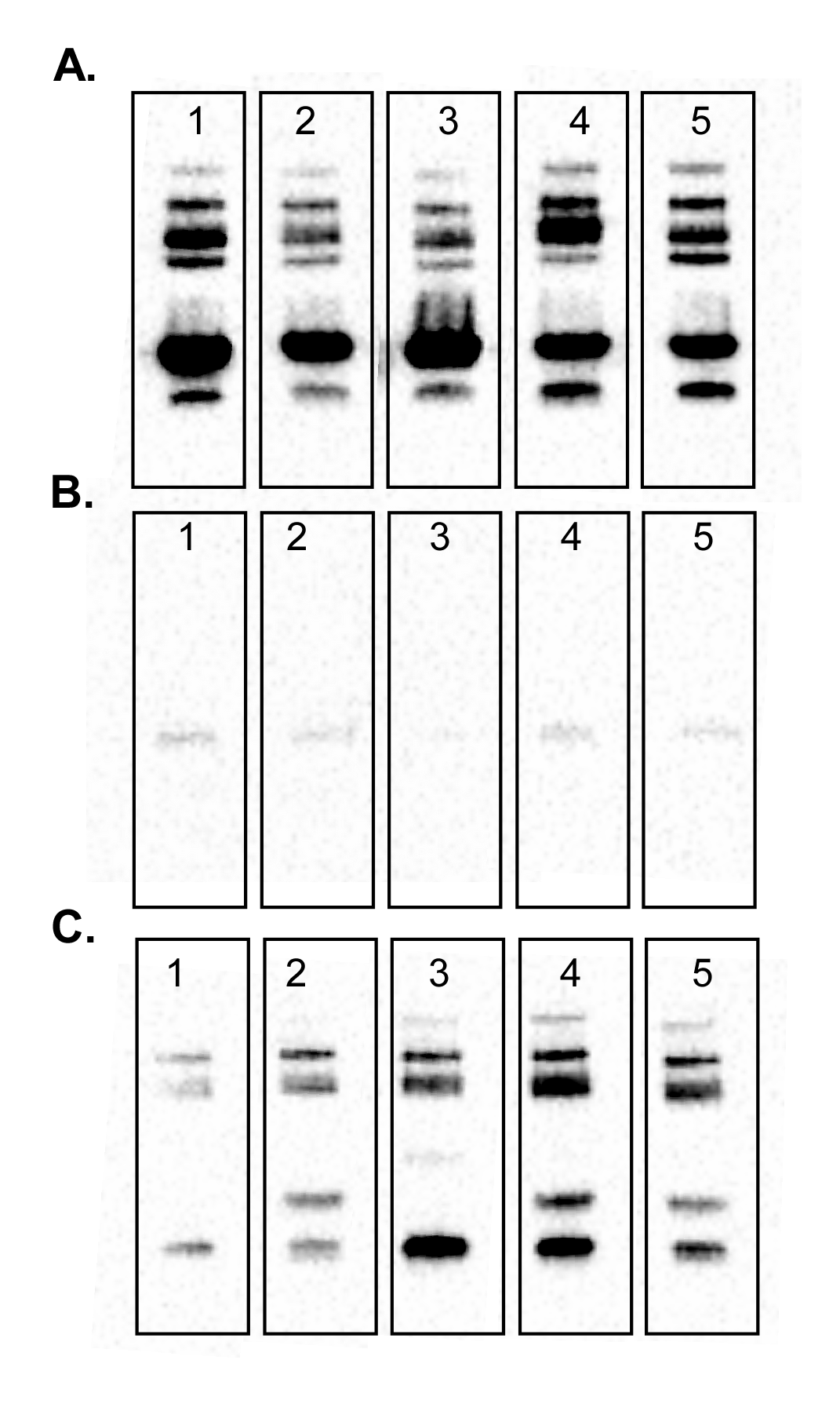

Supplement: S3 Fig — Seroconversion to inoculation with B. burgdorferi 297. Whole cell lysates from 297 wild-type strain blotted separately against serum collected 23 days post inoculation with wild-type (A), ΔdksA (B), or ΔdksA cDksA strain (C). Each blot was developed and imaged at the same time to control for differences in exposure. (TIF) [file ppat.1009072.s003.tif]

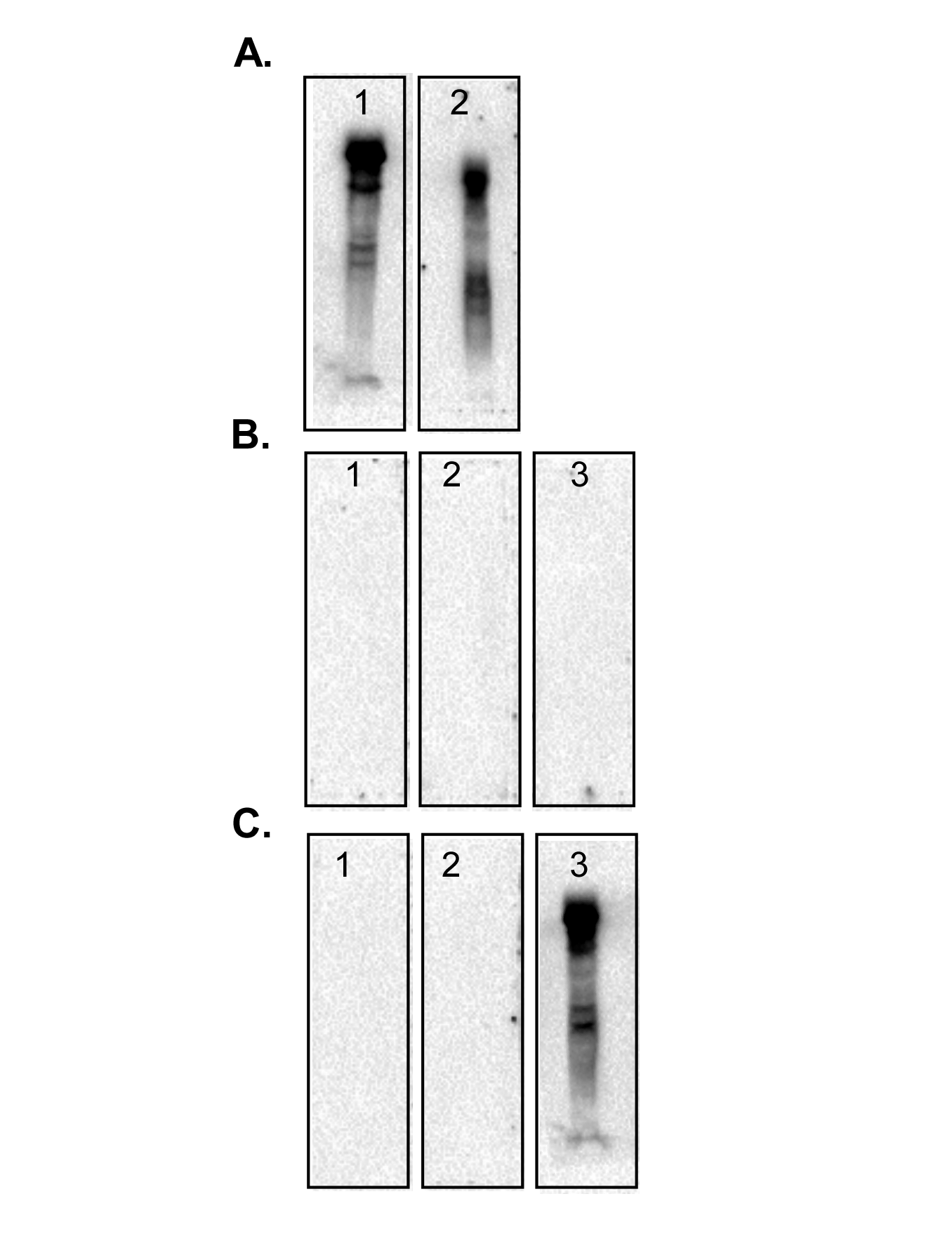

Supplement: S5 Fig — Whole cell lysates from 297 wild-type strain blotted separately against serum collected 3 weeks post-feeding by I. scapularis infected with wild-type (A), ΔdksA (B), or ΔdksA cDksA strain (C). Each blot was developed and imaged at the same time to control for differences in exposure. (TIF) [file ppat.1009072.s005.tif]

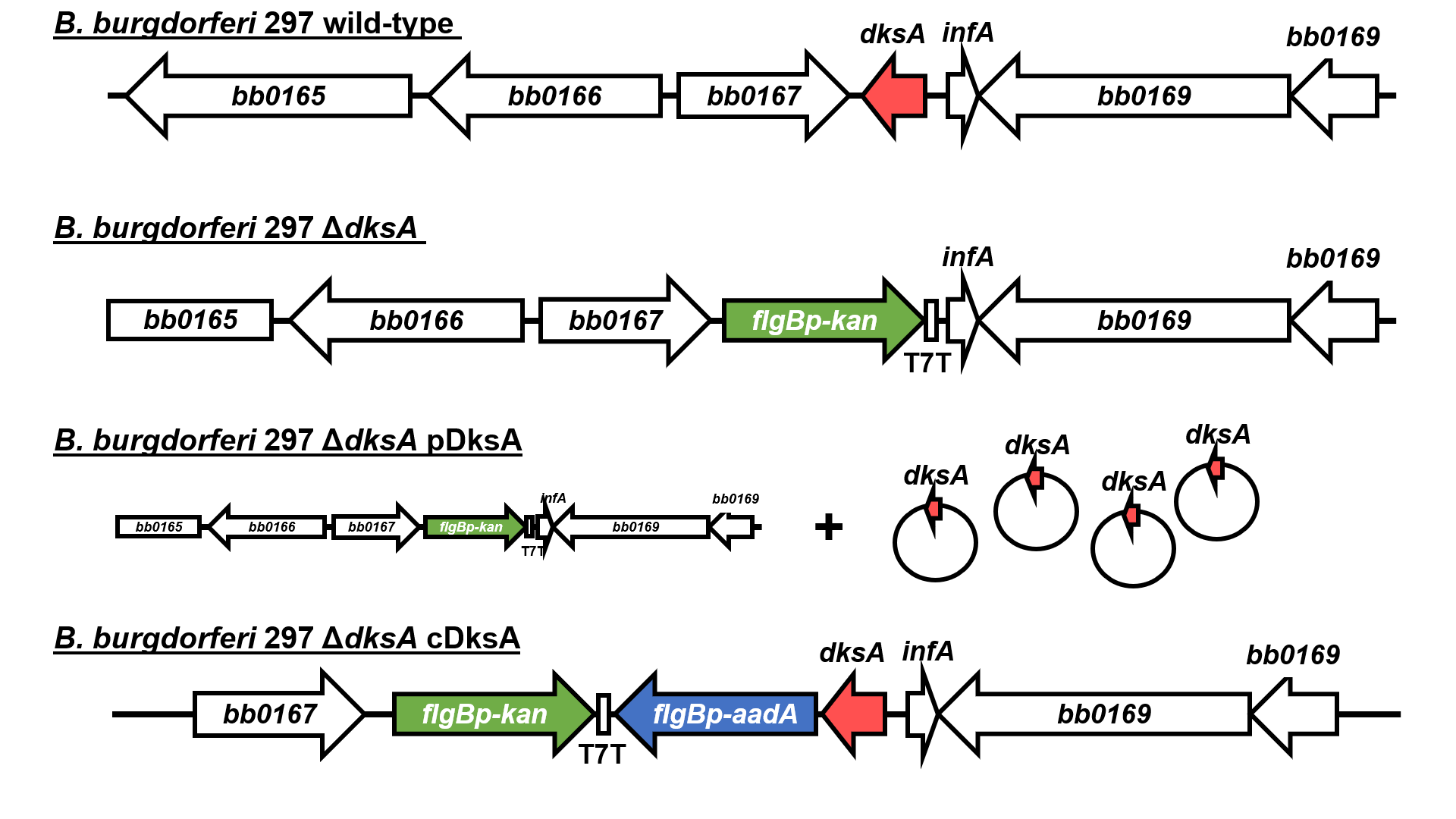

Supplement: S6 Fig — The pDksA strain over expresses the dksA gene, potentially due to dksA encoded on a multi-copy plasmid. To limit over expression of dksA, a single copy of the wild-type dksA allele was introduced into the genome of the ΔdksA strain. (TIF) [file ppat.1009072.s006.tif]
