# Supplementary material for: DksA-dependent regulation of RpoS contributes to Borrelia burgdorferi tick-borne transmission and mammalian infectivity
Source: PLoS Pathog. 2021 Feb 18;17(2):e1009072. doi: 10.1371/journal.ppat.1009072 (PMC7924775; doi:10.1371/journal.ppat.1009072)
Supplement: S1 Text — (DOCX) [file ppat.1009072.s008.docx]

**S1 Supplemental Methods**

***E. coli* strains and plasmids.**

The generation of the ∆*dksA* *E. coli* strain was carried out using the one-step, λ-Red-mediated gene replacement method of Datsenko and Wanner [1]. Briefly, primers encoding 30 nucleotides homologous to the *dksA* target gene followed by 20 nucleotides homologous to the pKD13 template plasmid were used for PCR amplification of the Flp recombinant target (FRT)-flanked kanamycin resistance cassette. The resulting PCR products were DpnI digested and electroporated into the competent *E. coli* BW25113 strain containing the plasmid pKD46, which expresses an isopropyl-β-thiogalactopyranoside-inducible λ-Red recombinase enzyme. The temperature sensitive pKD46 plasmid was subsequently cured by incubation of the strain at 37°C. A nonpolar deletion of the *E. coli dksA* was generated by recombining the two FRT sites flanking the kanamycin resistance cassette with the Flp recombinase encoded by the pCP20 plasmid [2]. The mutations were confirmed by PCR analysis.

The *dksA_bb_* allele was amplified by PCR using primers encoding NdeI and XhoI restriction sites (S1 Table), LaTaq polymerase (Takara, Mountain View, CA), and genomic DNA from *B. burgdorferi* strain B31 A3 as a template. The PCR product was inserted into the pBAD/HisA multiple cloning site using the indicated restriction enzymes and subsequent ligation. Resulting constructs were under the control of an L-arabinose-inducible pBAD promoter.

***E. coli* growth in minimal media.**

*E. coli* strains grown overnight in Luria-Bertani medium at 37˚C with shaking were subcultured 1:500 in N salts medium [5 mM KCl, 7.5 mM (NH_4_)SO_4_, 0.5 mM K_2_SO_4_, 1 mM KH_2_PO_4_, 38 mM glycerol, 0.1% Casamino Acids] supplemented with 0.5 mM MgCl_2_ and 0.02% L-arabinose. Diluted cultures were seeded in 96 well plates and grown at 37 °C with shaking at 282 r.p.m. with the optical density measured at 600 nm (OD_600nm_) using a Cytation 5 multi-mode plate reader (BioTek, Winooski, VT). Experiments were performed in triplicate.

***In vitro* susceptibility of *E. coli* to H_2_O_2_**

Overnight cultures of *E. coli* strains were diluted in N salts medium supplemented with 0.5 mM MgCl_2_, 0.1% casamino acids, and 0.02% L-arabinose to a cell density of ~5 x 10^5^ cells ml^-1^. One ml aliquots of cell suspensions were transferred to 5 ml polypropylene culture tubes and incubated in the presence or absence of 1 mM H_2_O_2_ for 2 h at 37°C. Following incubation, serial dilutions prepared for each culture were plated on LB agar, incubated overnight at 37°C, and colony forming units (CFU) enumerated. Percent survival was calculated as (CFU t_2h_/CFU t_0h_) x 100.

**Real-time *in vitro* transcription assay**

*In vitro* transcription was monitored real-time using fluorescence. Reactions were prepared in 50 µl volumes in MicroAmp optical 96-well reaction plates. Transcripts from the *E. coli* promoter *rrnB* P1 were detected using a molecular beacon (5´-FAM-CGCUUUUUUUUUUUUGCG-DABCYL-3´, with a 2´-O-methylribonucleotide backbone). The genomic DNA sequence containing -200 to +200 around the *rrnB* P1 site was amplified by PCR using primers incorporating a molecular beacon base pairing sequence (5´-AAAAAAAAAAAA-3´) downstream end from the promoter site. The amplified DNA material was cleaned with a DNA cleanup kit prior to addition an *in vitro* transcription reaction as the double stranded DNA template. Each reaction contained 1X reaction buffer, 200 µM NTP, 0.8U RNase inhibitor, 500 nM molecular beacon, and 1 unit of RNA polymerase holoenzyme (New England Biolabs, Ipswich, MA, United States). DksA and ppGpp were mixed with the reaction solution prior to transcription initiation by the addition of DNA template to 10 nM final concentration. fluorescence (ex 495 nm, em 515 nm) of the reaction mixture was determined (every 30 s for 30 min, 37°C) was determined using an ABI 7500 real-time PCR machine (Applied Biosystems, Foster City, California, United States). Raw fluorescence values were plotted using GraphPad Prism software. Replicate experiments were performed using altered plate layout to control for possible differences in fluorescence sensitivity by well locations.

1. Datsenko KA, Wanner BL. One-step inactivation of chromosomal genes in Escherichia coli K-12 using PCR products. Proc Natl Acad Sci U S A. 2000;97(12):6640-5.

2. Cherepanov PP, Wackernagel W. Gene disruption in Escherichia coli: TcR and KmR cassettes with the option of Flp-catalyzed excision of the antibiotic-resistance determinant. Gene. 1995;158(1):9-14.
