# Supplementary material for: DksA-dependent regulation of RpoS contributes to Borrelia burgdorferi tick-borne transmission and mammalian infectivity
Source: PLoS Pathog. 2021 Feb 18;17(2):e1009072. doi: 10.1371/journal.ppat.1009072 (PMC7924775; doi:10.1371/journal.ppat.1009072)

**Original, uncropped Western blots, gel stains, and phosphor screen images.** Red squares indicate cropped section selected for the final figures. Gels without cropping were used for quantitative analysis displayed in the original figure.


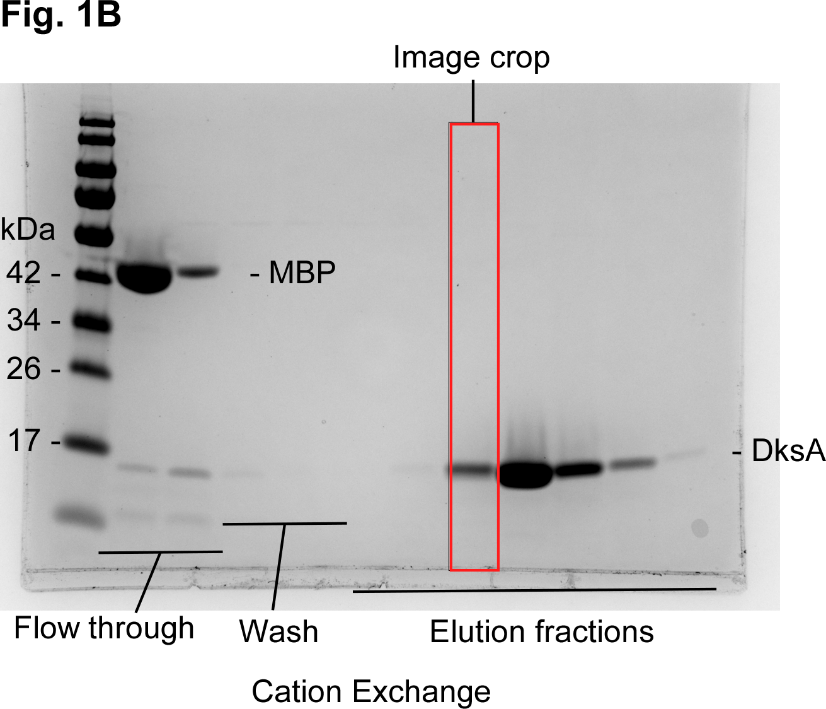


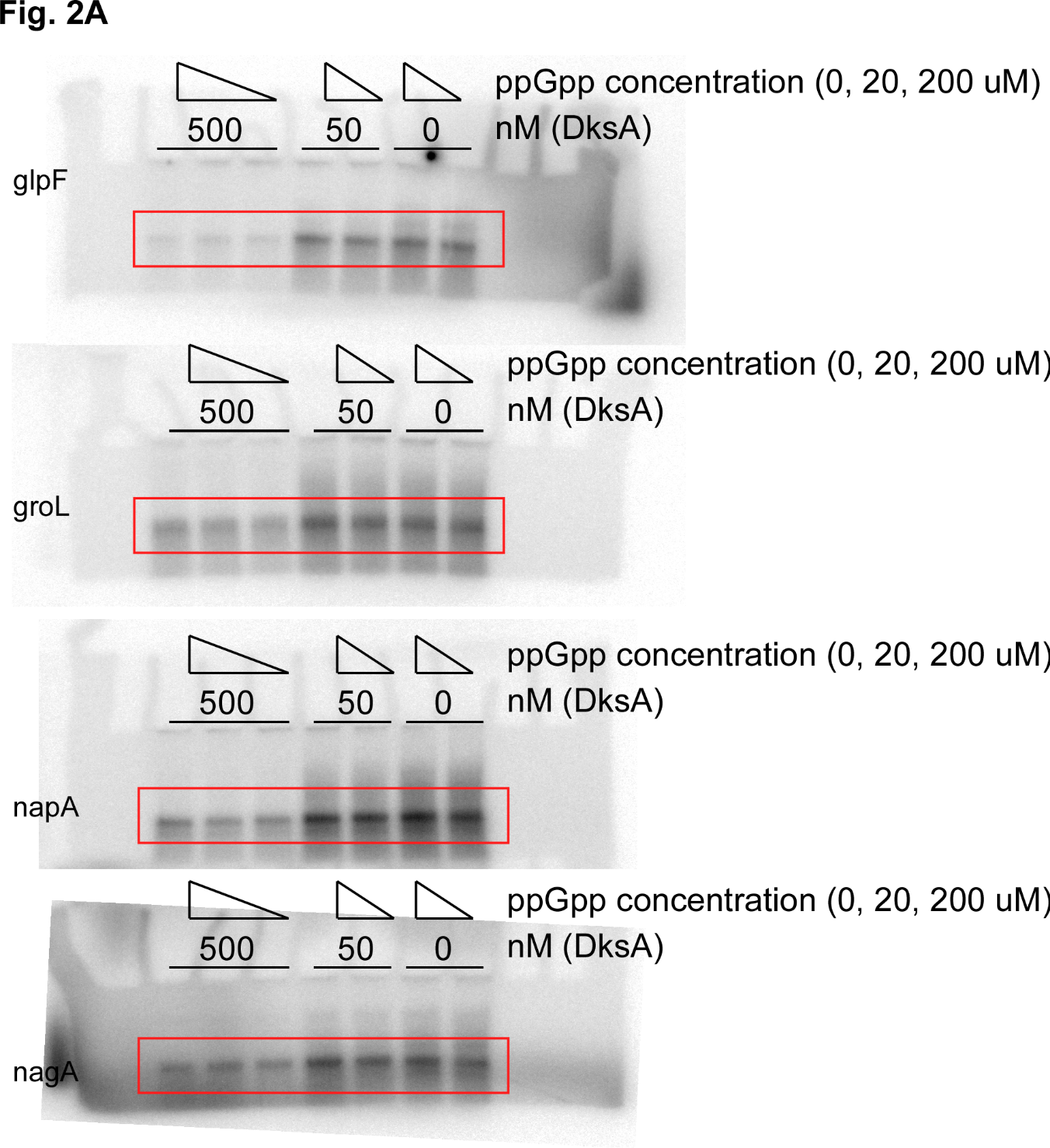

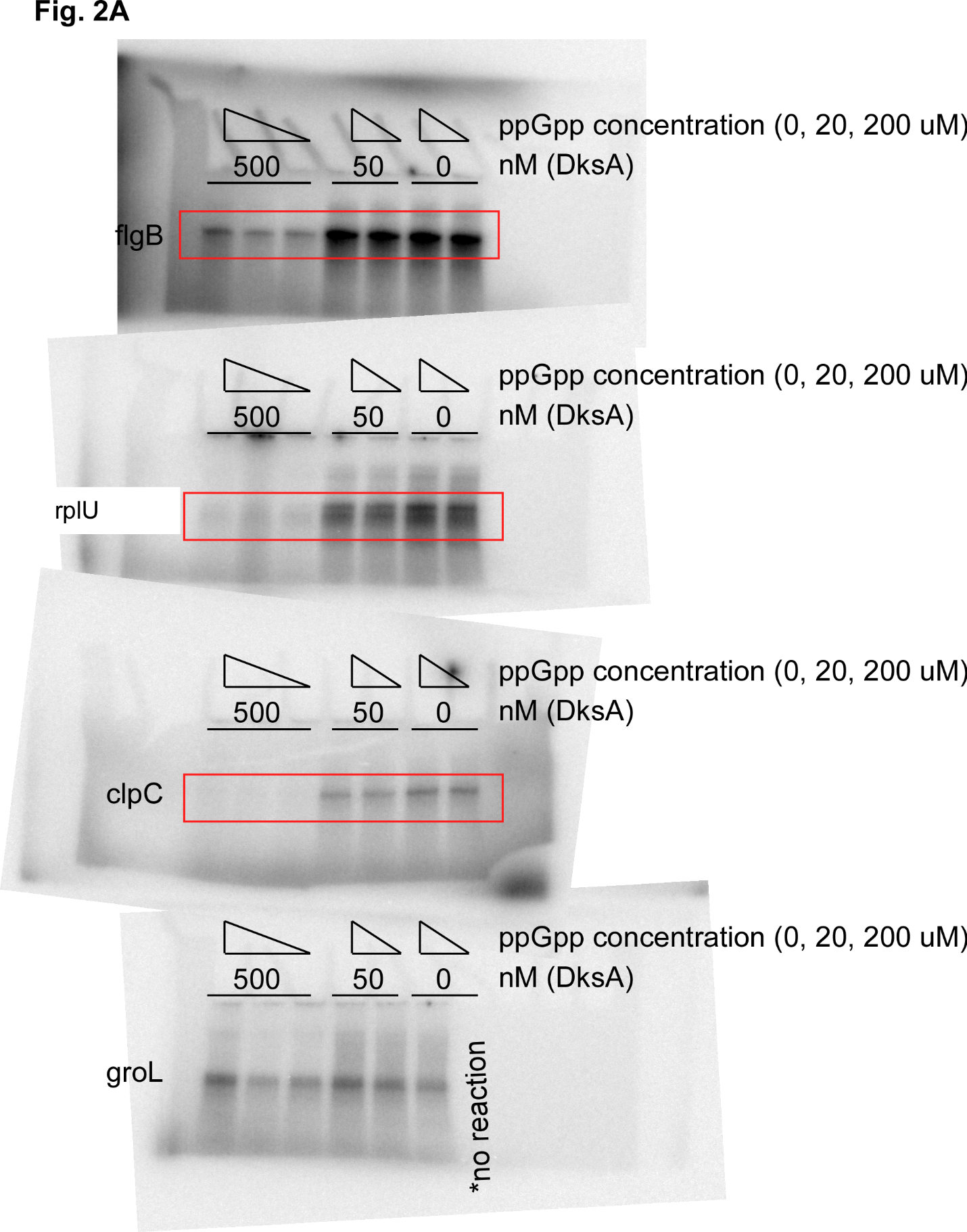

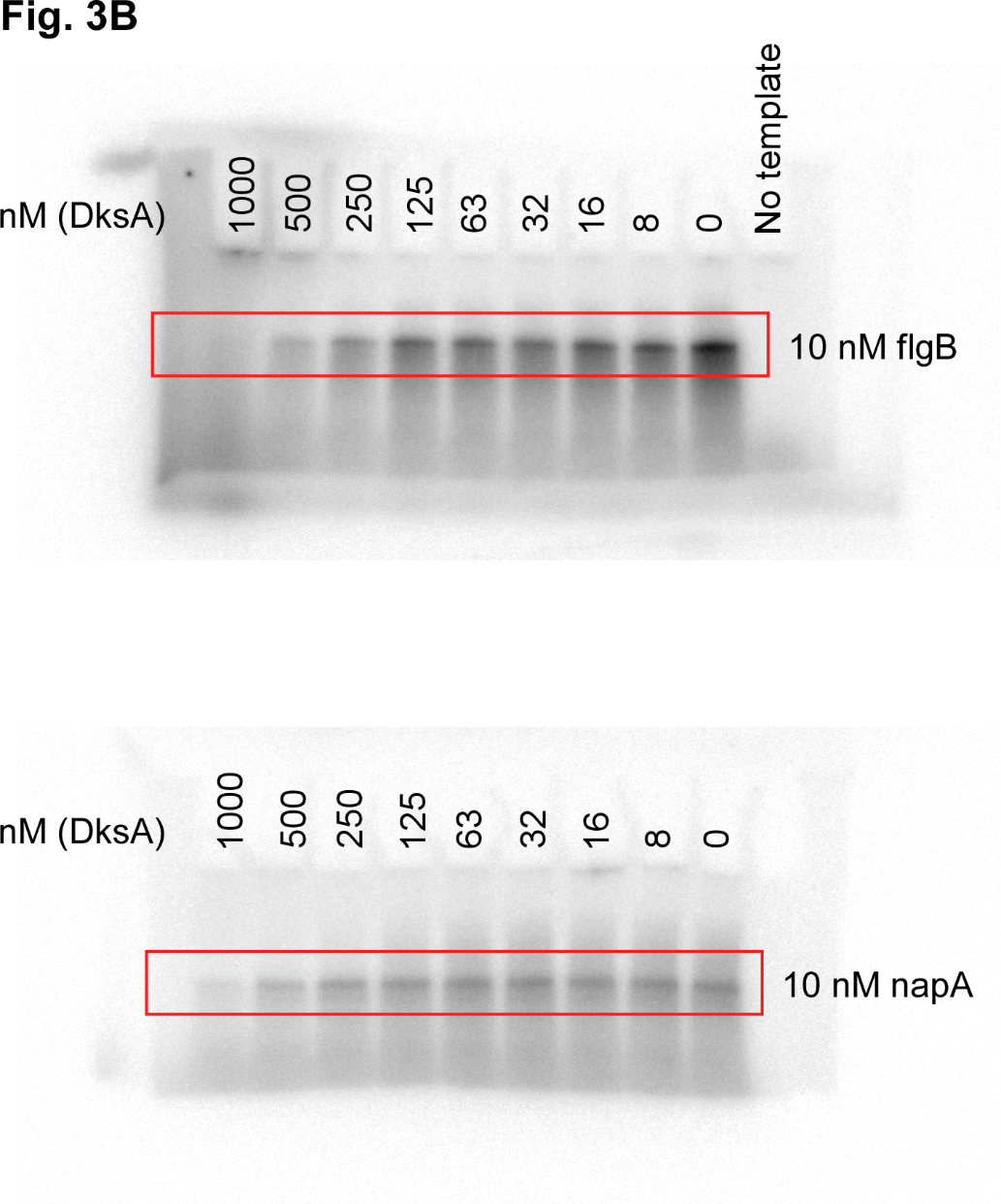

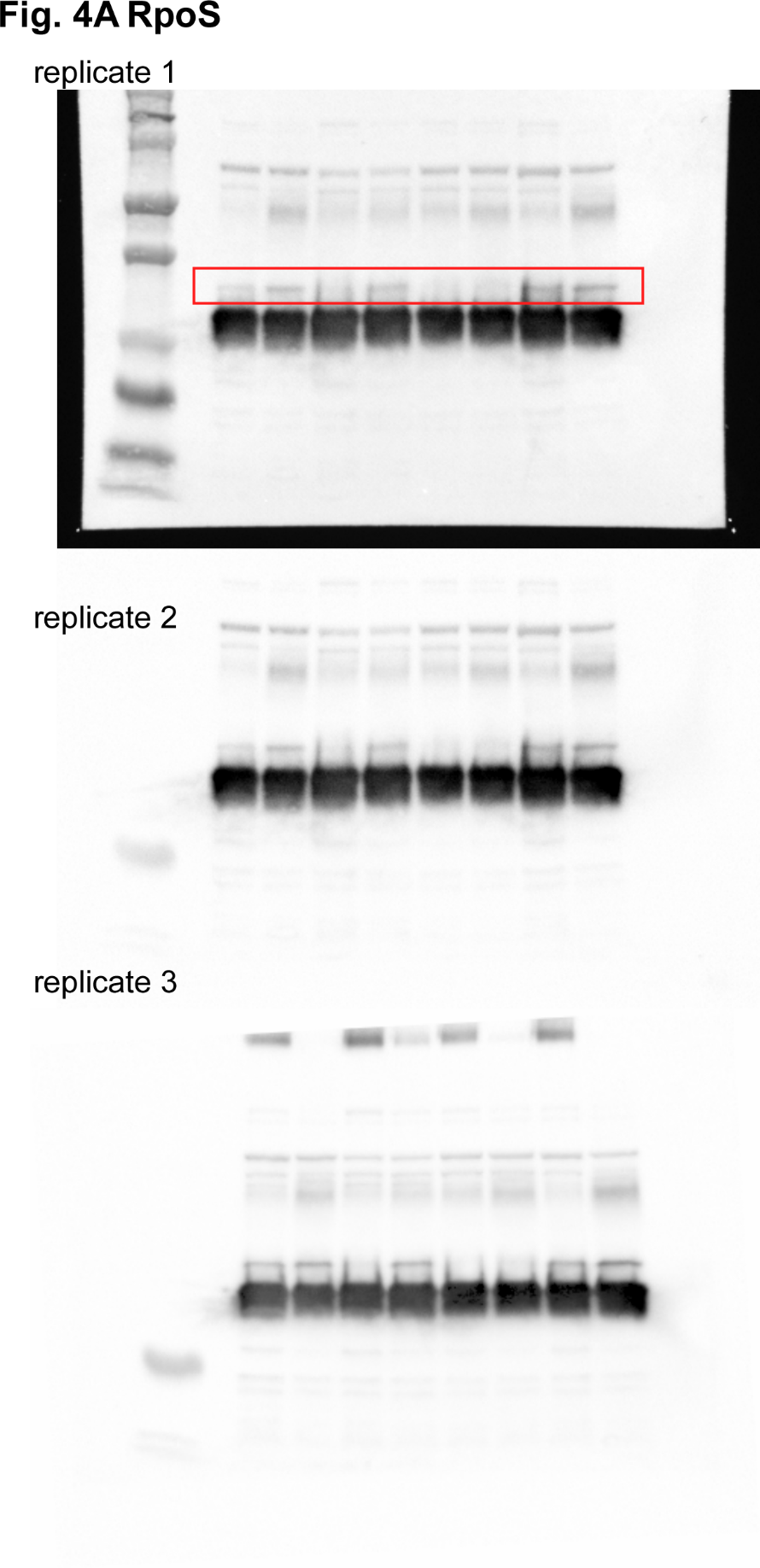

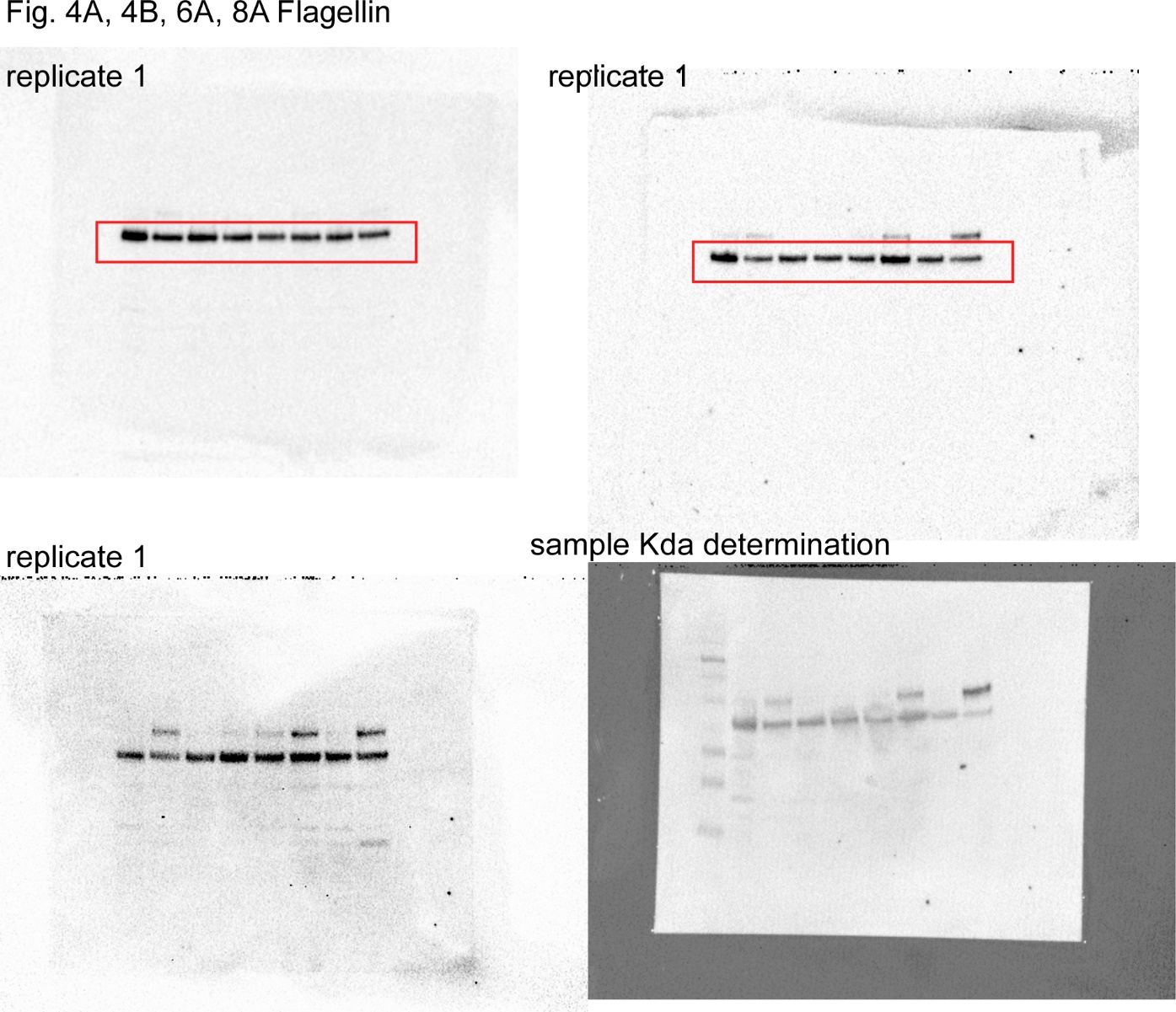

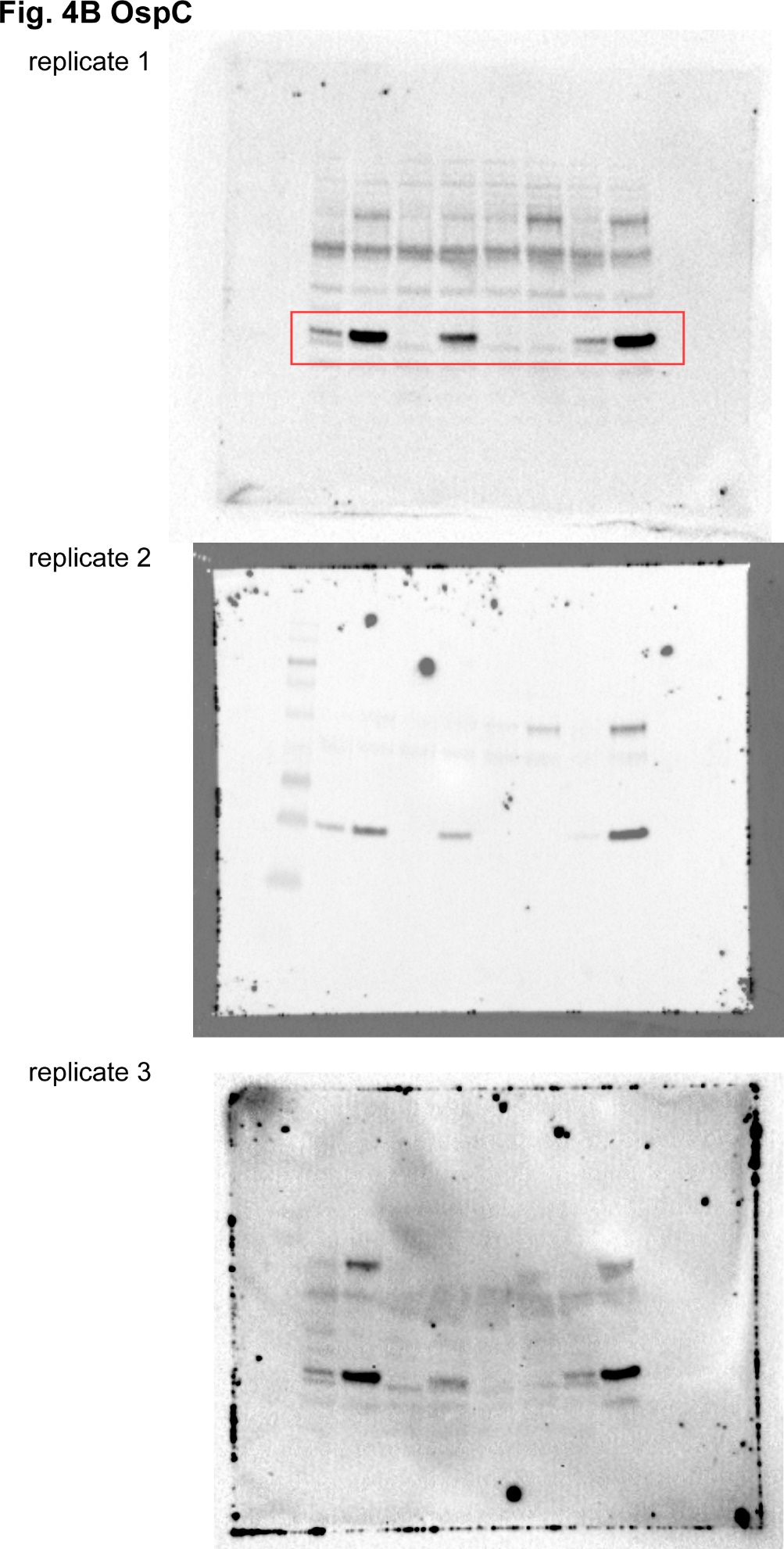

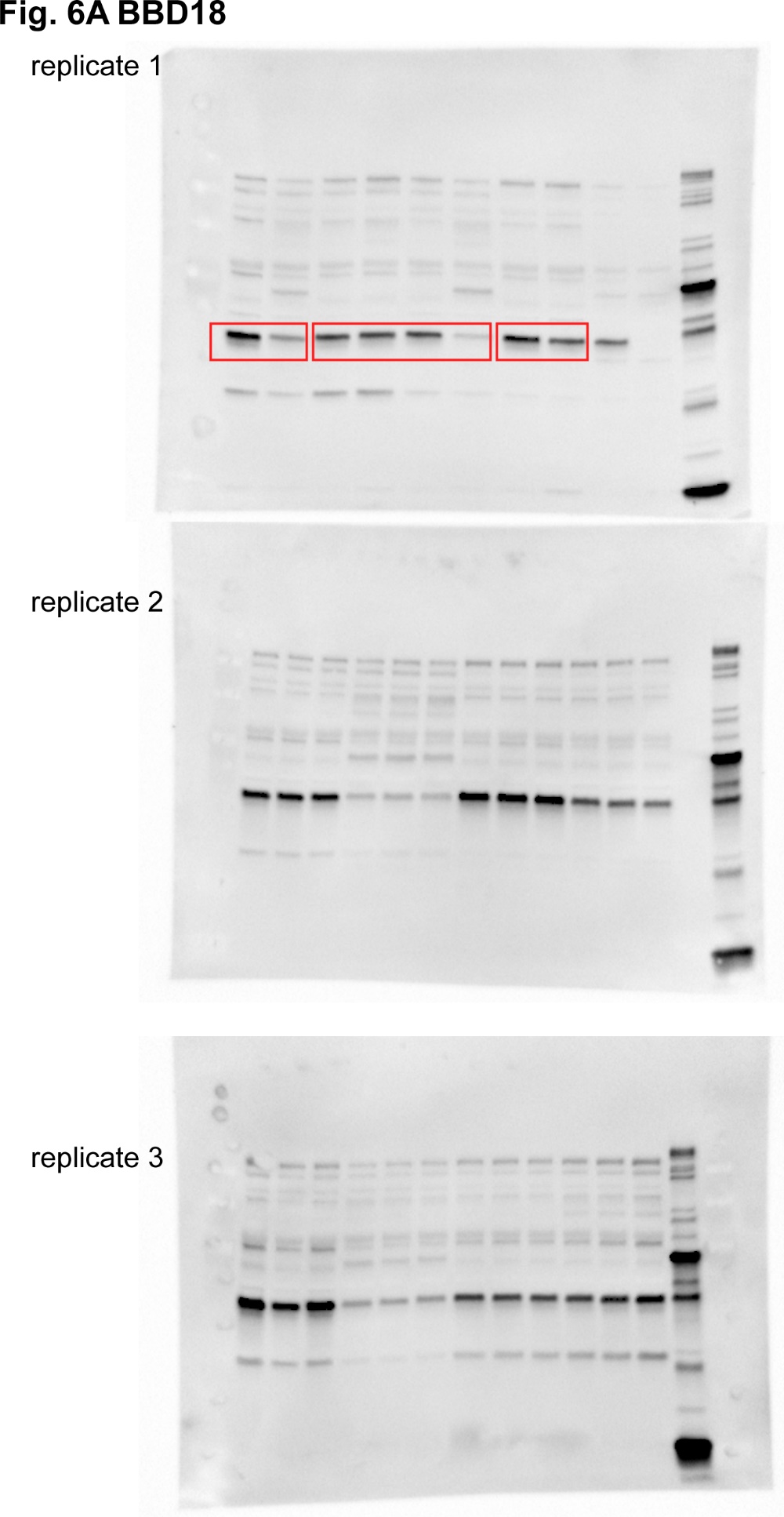

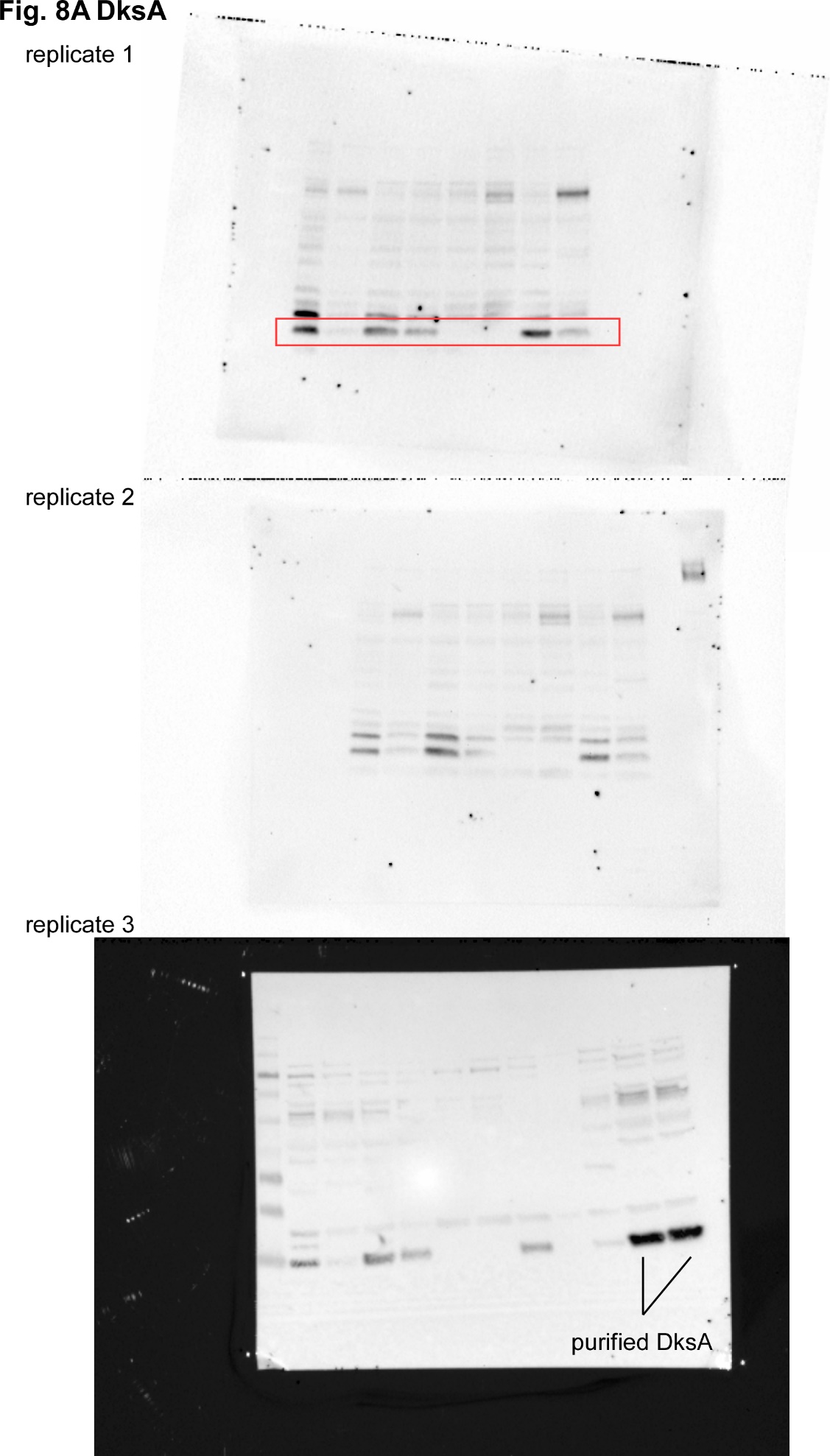

Supplement: S2 Text — Red squares indicate cropped section selected for the final figures. Gels without cropping were used for quantitative analysis displayed in the original figure. (DOCX) [file ppat.1009072.s009.docx]
